# Identification of Genes Required for Nonhost Resistance to *Xanthomonas oryzae* pv. *oryzae* Reveals Novel Signaling Components

**DOI:** 10.1371/journal.pone.0042796

**Published:** 2012-08-13

**Authors:** Wen Li, You-Ping Xu, Zhi-Xin Zhang, Wen-Yuan Cao, Fei Li, Xueping Zhou, Gong-You Chen, Xin-Zhong Cai

**Affiliations:** 1 Institute of Biotechnology, College of Agriculture and Biotechnology, Zhejiang University, Hangzhou, China; 2 Center of Analysis and Measurement, Zhejiang University, Hangzhou, China; 3 State Key Laboratory of Rice Biology, Zhejiang University, Hangzhou, China; 4 School of Agriculture and Biology, Shanghai Jiaotong University/Key Laboratory of Urban (South) by Ministry of Agriculture, Shanghai, China; University of Wisconsin-Milwaukee, United States of America

## Abstract

**Background:**

Nonhost resistance is a generalized, durable, broad-spectrum resistance exhibited by plant species to a wide variety of microbial pathogens. Although nonhost resistance is an attractive breeding strategy, the molecular basis of this form of resistance remains unclear for many plant-microbe pathosystems, including interactions with the bacterial pathogen of rice, *Xanthomonas oryzae* pv. *oryzae* (Xoo).

**Methods and Findings:**

Virus-induced gene silencing (VIGS) and an assay to detect the hypersensitive response (HR) were used to screen for genes required for nonhost resistance to Xoo in *N. benthamiana*. When infiltrated with Xoo strain YN-1, *N. benthamiana* plants exhibited a strong necrosis within 24 h and produced a large amount of H_2_O_2_ in the infiltrated area. Expression of HR- and defense-related genes was induced, whereas bacterial numbers dramatically decreased during necrosis. VIGS of 45 *ACE* (Avr/Cf-elicited) genes revealed identified seven genes required for nonhost resistance to Xoo in *N. benthamiana*. The seven genes encoded a calreticulin protein (ACE35), an ERF transcriptional factor (ACE43), a novel Solanaceous protein (ACE80), a hydrolase (ACE117), a peroxidase (ACE175) and two proteins with unknown function (ACE95 and ACE112). The results indicate that oxidative burst and calcium-dependent signaling pathways play an important role in nonhost resistance to Xoo. VIGS analysis further revealed that *ACE35*, *ACE80*, *ACE95* and *ACE175*, but not the other three *ACE* genes, interfered with the Cf-4/Avr4-dependent HR.

**Conclusions/Significance:**

*N. benthamiana* plants inoculated with Xoo respond by rapidly eliciting an HR and nonhost resistance. The oxidative burst and other signaling pathways are pivotal in Xoo-*N. benthamiana* nonhost resistance, and genes involved in this response partially overlap with those involved in Cf/Avr4-dependent HR. The seven genes required for *N. benthamiana*-mediated resistance to Xoo provide a basis for further dissecting the molecular mechanism of nonhost resistance.

## Introduction

It is a general phenomenon that microbial pathogens can successfully infect only a limited number of plant species. When non-adapted pathogens attempt to colonize plant species outside of the normal host range, nonhost disease resistance is triggered. Nonhost resistance is durable and broad-spectrum, which makes it highly desirable for mediating resistance to plant diseases [1].

Substantial research has been dedicated to understand the molecular basis of nonhost resistance. It has been established that nonhost resistance to non-adapted pathogens results from both preformed and induced defence mechanisms [2]–[10]. Constitutive defence mechanisms are more likely to contribute to nonhost resistance to pathogens of diverse plant families than to pathogens of plants closely related to the nonhost [9]. The host range of a given pathogen depends on its effector repertoire [8]–[10], and varies from very narrow (e.g. one to several closely-related plant species) to very broad (many species in different plant families). It is now apparent that nonhost resistance to non-adapted pathogens includes defense mechanisms similar to those utilized for adapted pathogens, including pathogen-associated molecular pattern (PAMP)- and effector-triggered immunity (PTI and ETI, respectively) [8]–[10]. As the phylogenetic distance between plants increases, PTI plays a greater role in nonhost resistance than ETI [10]. Efforts to dissect the molecular mechanisms of nonhost resistance using genetic approaches have resulted in the identification of essential regulatory genes for nonhost resistance, primarily from *Arabidopsis* [8]–[10].

Bacterial blight caused by *Xanthomonas oryzae* pv. *oryzae* (Xoo) is a devastating disease of rice (*Oryza sativa*), which is a staple food in many countries and a model plant for cereal biology [11]. Significant progress has been made in understanding the rice–Xoo interaction [12]. Collections of Xoo effectors have been identified, including a group of transcription activator-like (TAL) type III effectors [13]–[15]. Both ETI- and PTI-mediated resistance have been identified in the rice-Xoo pathosystem. For ETI, genes involved in rice disease resistance (*R*) (also known as *Xa* genes) and cognate Xoo Avr effectors have been cloned and functionally analysed; a representative pair is *Xa27* and *AvrXa27* [12]. PTI in the rice - Xoo interaction is exemplified by *Xa21* and *Ax21* [16]–[17]. Xa21 was originally cloned as an *R* gene that conferred ETI [18]–[19], but was later found to be a pattern recognition receptor (PRR) that triggers PTI by recognizing Ax21, which exists in all sequenced genomes of *Xanthomonas* and in *Xylella* [20]. Identification of XA21-binding (XB) proteins, e.g. XB3 (E3 ubiquitin ligase), XB10 (OsWRKY62), XB15 (PP2C phosphatase) and XB24 (ATPase), has provided significant insight into the regulation of XA21-mediated PTI [21]–[24].

It is well-established that nonhost resistance to Xoo requires a functional type III secretion system for effector delivery into plant cells. The type III secretion system contains hypersensitive response (HR) and pathogenicity (hrp) genes, which are essential both for bacterial pathogenesis in susceptible hosts and for induction of the HR in resistant host and nonhost plants [25]. Regulation of *hrp* gene expression in Xoo is complex and typically requires two key regulatory proteins, HrpG and HrpX [15], [26]. In contrast to the pathogen part of the interaction, few studies have addressed the basis of nonhost resistance to Xoo. A limited number of host components essential to nonhost resistance have been reported. For *Xanthomonas* spp., clues can be derived from nonhost resistance in maize to *X. oryzae* pv. *oryzicola* (Xoc), a pathovar closely related to Xoo. For example, *avrRxo1* from Xoc induces a nonhost defense reaction on maize containing the resistance gene *Rxo1* [27], and *Rxo1* confers resistance to Xoc in rice as well [28]. It is unclear whether similar mechanisms exist for nonhost resistance to Xoo.

In this study, we describe an efficient screen for plant genes required for nonhost resistance to Xoo; the screen is based on functional analysis by virus-induced gene silencing (VIGS) and HR detection assays. A group of genes were identified using this approach and suggest the involvement of reactive oxygen species (ROS) accumulation and calcium-dependent signaling pathways in nonhost resistance.

## Results

### Strong Necrosis is Induced in *N. benthamiana* Plants Infiltrated with Xoo

**Figure 1:**
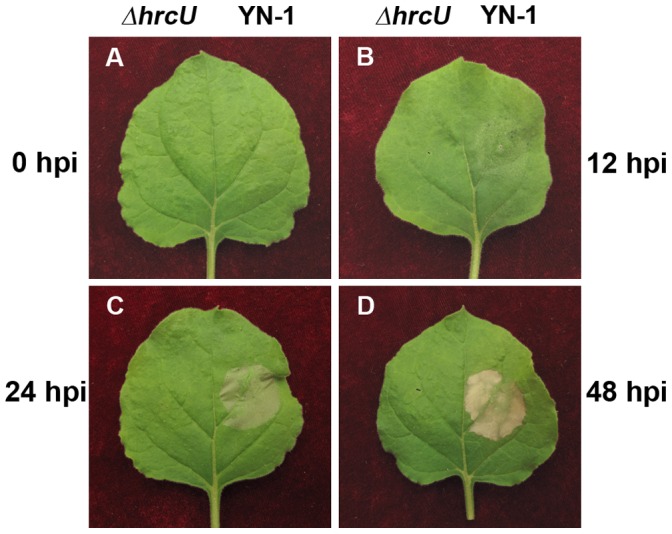
Necrosis symptoms in *Nicotiana benthamiana* leaves infiltrated with *Xanthomonas oryzae* pv. *oryzae* (Xoo) and a *hrcU* mutant. Suspension of Xoo YN-1 and a *hrcU* mutant (Δ*hrcU*) at 8×10^7^ cfu/ml was infiltrated into right and left half of leaves of *N. benthamiana* plants, respectively. Symptoms at 0, 12, 24 and 48 hours post infiltration (hpi) were shown.

Generally, Xoo is considered a non-adaptive pathogen with respect to *N. benthamiana*; however, its ability to induce an HR in this plant species is strain-dependent [29]. To assess the interaction of *N. benthamiana* with Xoo strain YN-1, bacterial cells were infiltrated into leaves, and the reaction was monitored. The infiltrated areas lost vigor and showed signs of wilting at 12 h post-infiltration (hpi) (Figure 1B); formed a dark green to grey necrosis at 24 hpi (Figure 1C), which turned into brown within 48 hpi (Figure 1D). The necrosis was restricted to the infiltrated areas, and the necrotic tissue was latter desiccated and brittle (Figure 1). These characteristics are reminiscent of a strong hypersensitive necrosis. A *hrcU* mutant (Δ*hrcU*) lost the ability to induce an HR (Figure 1, left half of leaves), demonstrating that *HrcU* is required for YN-1 elicitation of hypersensitive necrosis in *N. benthamiana*.

### H_2_O_2_ but not O_2_^⋅−^ Accumulates Prior to the Appearance of Xoo-induced Hypersensitive Necrosis

**Figure 2:**
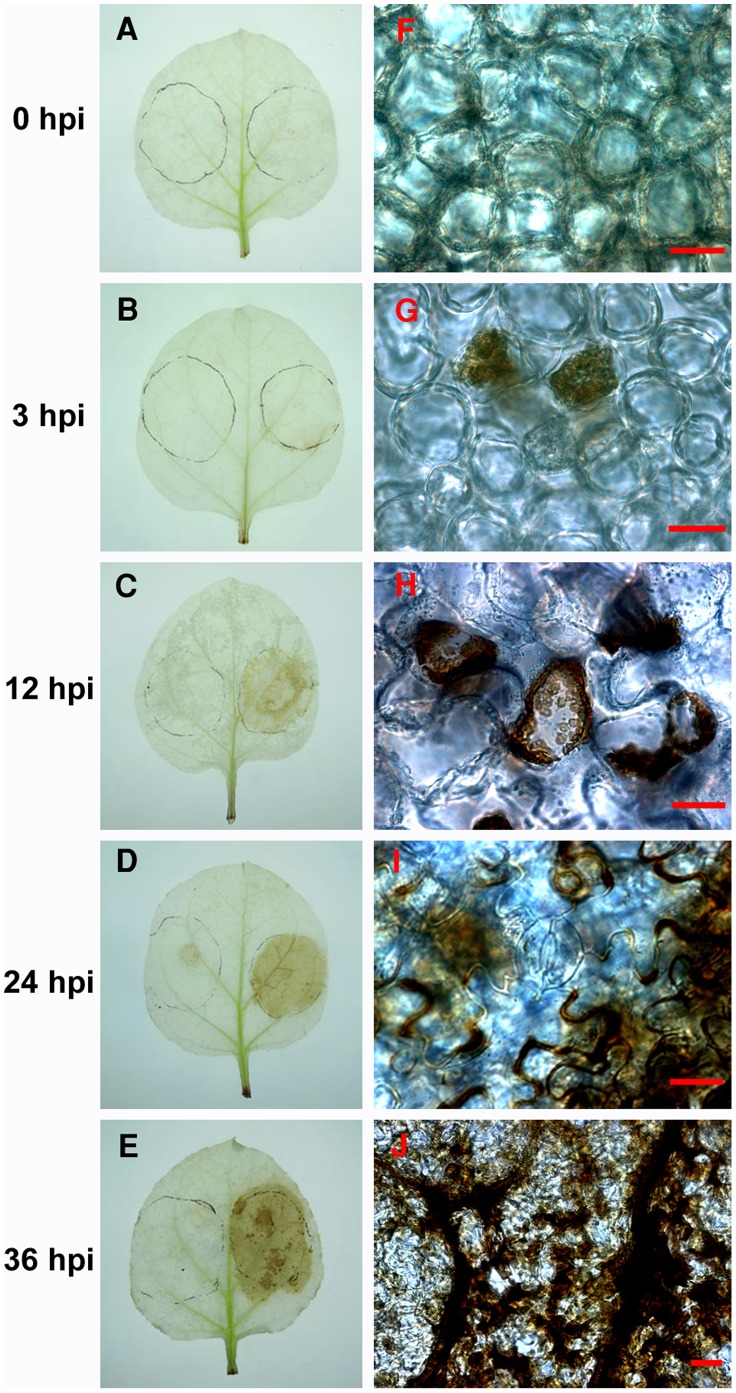
Strong H_2_O_2_ accumulation in Xoo-infiltrated area prior to formation of hypersensitive necrosis. Leaves that were infiltrated with Xoo (right half) and *hrcU* mutant (left half) suspension were sampled sequentially at different time-points. H_2_O_2_ in leaves was detected by diaminobenzidine (DAB) staining analysis, and appeared as brown deposit. Bar = 20 µm.

The HR is frequently accompanied by accumulation of reactive oxygen species (ROS). Thus, the production of H_2_O_2_, one of the primary species of ROS, was analyzed in Xoo-infiltrated *N. benthamiana* leaves by diaminobenzidine (DAB) staining. In Δ*hrcU*-infiltrated leaves, no staining was observed (Figure 2A–E, left half of leaves). In YN-1-infiltrated leaves, some tissues near the vasculature stained brownish at 3 hpi (Figure 2B, right half of leaves). With time, the stained area extended and deepened in color; at 12 hpi, the entire infiltrated area was stained (Figure 2C–E right half of leaves), indicating extensive accumulation of H_2_O_2_. To examine H_2_O_2_ accumulation at the cellular level, the tissues of the stained leaves were subjected to microscopic analysis. Some mesophyll cells stained brownish as early as 3 hpi (Figure 2G). Beginning at 6 hpi, the number of stained cells increased significantly. At 12 hpi, stained cells showed a deep brown color, indicating strong accumulation of H_2_O_2_, and contained granules that also stained brown. The walls of some stained cells were broken, resulting in an outflow of granules (Figure 2H). At 24 hpi, most of the stained mesophyll cells collapsed and the epidermal cells were also deeply stained (Figure 2I); at 36 hpi, the guard cells were stained and both epidermal and mesophyll cells collapsed (Figure 2J). These data revealed that H_2_O_2_ accumulation occurred before hypersensitive necrosis was visible.

The superoxide anion (O_2_^⋅−^) is another ROS that is commonly produced during the HR. Interestingly, nitro blue tetrazolium (NBT) staining for O_2_^⋅−^ revealed no significant change in O_2_^⋅−^ production in *N. benthamiana* leaves infiltrated with YN-1 or the Δ*hrcU* mutant (data not shown). These results indicated that H_2_O_2_ but not O_2_^⋅−^ contributes to Xoo-induced hypersensitive necrosis.

### Induction of HR- and Defense-related Genes in Xoo-infiltrated Leaves

**Figure 3:**
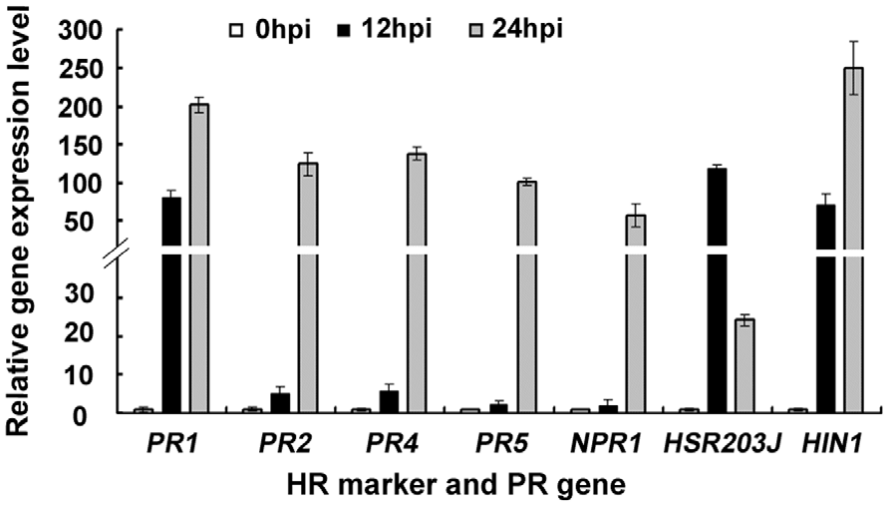
Upregulation of HR- and defense-related genes in Xoo-infiltrated leaves. Gene expression was analyzed by qRT-PCR with gene specific primers. HR marker genes *HIN1* and *HSR203J*, a pivatol defense regulator *NPR1* and a set of *PR* genes were analyzed for their expression in Xoo-infiltrated plants at 12 hpi and 24 hpi.

To understand the molecular response of *N. benthamiana* to Xoo during HR-associated necrosis, we monitored the expression of HR marker genes *HIN1* and *HSR203J*, the pivotal defense regulatory gene *NPR1*, and a set of pathogenesis-related (*PR*) genes by quantitative real-time PCR (qRT-PCR) and semi-quantitative reverse transcription PCR (RT-PCR). PCR results indicated that *HIN1* and *HSR203J* expression was induced 71- and 119-fold, respectively, at 12 hpi, which is before necrosis was macroscopically visible. At 24 hpi, when strong visible necrosis was observed, *HIN1* was further strongly up-regulated (250-fold) whereas *HRS203J* expression dropped to 24-fold as compared to expression in noninfiltrated control leaves. Expression of *NPR1* was weakly induced at 12 hpi but strongly up-regulated (57-fold) at 24 hpi. The *PR* genes displayed differential expression patterns. *PR1* was expressed at high levels in water-infiltrated control leaves and was strongly up-regulated in Xoo-infiltrated leaves (81- and 202-fold induction at 12 and 24 hpi, respectively). *PR2* was expressed at a moderate level in water-infiltrated leaves and was up-regulated in Xoo-infiltrated leaves mildly at 12 hpi (5-fold induction) and strongly at 24 hpi (125-fold induction), respectively. Expression of *PR4* and *PR5* was not obvious in control leaves, but was mildly induced in Xoo-infiltrated leaves at 12 hpi and strongly induced at 24 hpi (100-fold induction) (Figure 3; Figure S1). These results support the hypothesis that the necrosis observed in Xoo-infiltrated leaves is a type of HR.

### Numbers of Xoo Cells Significantly Decrease during Necrosis

**Figure 4:**
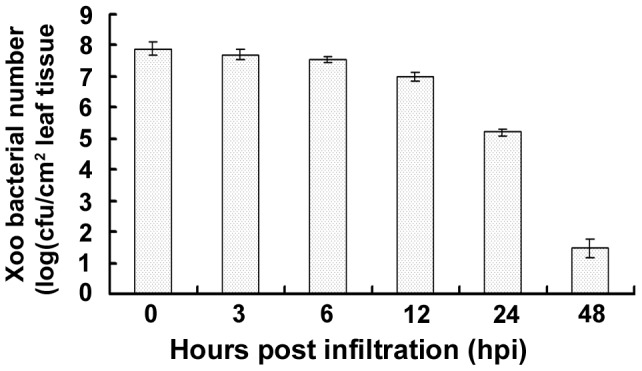
Population dynamics of Xoo inside the plant leaf tissues after infiltration. Cell numbers of Xoo in the infiltrated leaf tissues were determined based on calculation of colony-forming units of the sampled tissues.

To further investigate whether Xoo-induced necrosis in *N. benthamiana* is a form of HR, which is a hallmark of nonhost disease resistance, the number of bacterial cells in infiltrated leaves was analyzed. The results showed that the population dynamics of Xoo was correlated with the timing of necrosis (Figure 4). Bacterial numbers decreased slowly from 0–12 hpi before necrosis was observed; however, bacterial cell counts dropped dramatically when necrosis was readily visible, e.g. 2.8- and 6.4-fold reduction at 24 and 48 hpi, respectively (Figure 4). These results, together with the symptomology, cytology, and molecular data, demonstrate that *N. benthamiana* forms an HR and exhibits nonhost resistance in response to the non-adaptive pathogen Xoo YN-1.

### VIGS Screen to Identify Genes Required for the HR in the Xoo-*N. benthamiana* Interaction

Previously, we identified over 200 *ACE* (Avr/Cf-elicited) genes that displayed differential expression in tomato seedlings exhibiting a Cf-4/Avr4-dependent HR and in those not showing such a HR [30]–[31]. In the present study, 45 *ACE* genes, which are involved in defense, signal transduction, transcriptional regulation and metabolism, were selected for screening potential regulators of HR and nonhost resistance to Xoo in *N. benthamiana* employing VIGS (Table S1). Fragments of these *ACE* genes were cloned into the TRV silencing vector pYL156 and the HR was scored after Xoo infiltration into silenced plants. Phenotypes of the HR were assigned to one of three groups based on the intensity and percentage of cell death in infiltrated areas; e.g. full to nearly full HR; partial HR, and no HR (Figure S2). A full HR meant that tissues of the infiltrated region were completely dead and had collapsed (Figure S2, A, B). A nearly full HR signified that over 75% of the tissue in infiltrated areas was dead or collapsed (Figure S2, C). Phenotypes in the partial HR group showed less than 75% cell death or yellowing in the infiltrated area (Figure S2, D–F). The no HR phenotype did not exhibit cell death in the infiltrated area (Figure S2, G–I). Plants inoculated with the control vector (CV, containing a fragment of eGFP in pTRV2) exhibited a full HR or occasionally a nearly full HR in response to Xoo infiltration.

**Figure 5:**
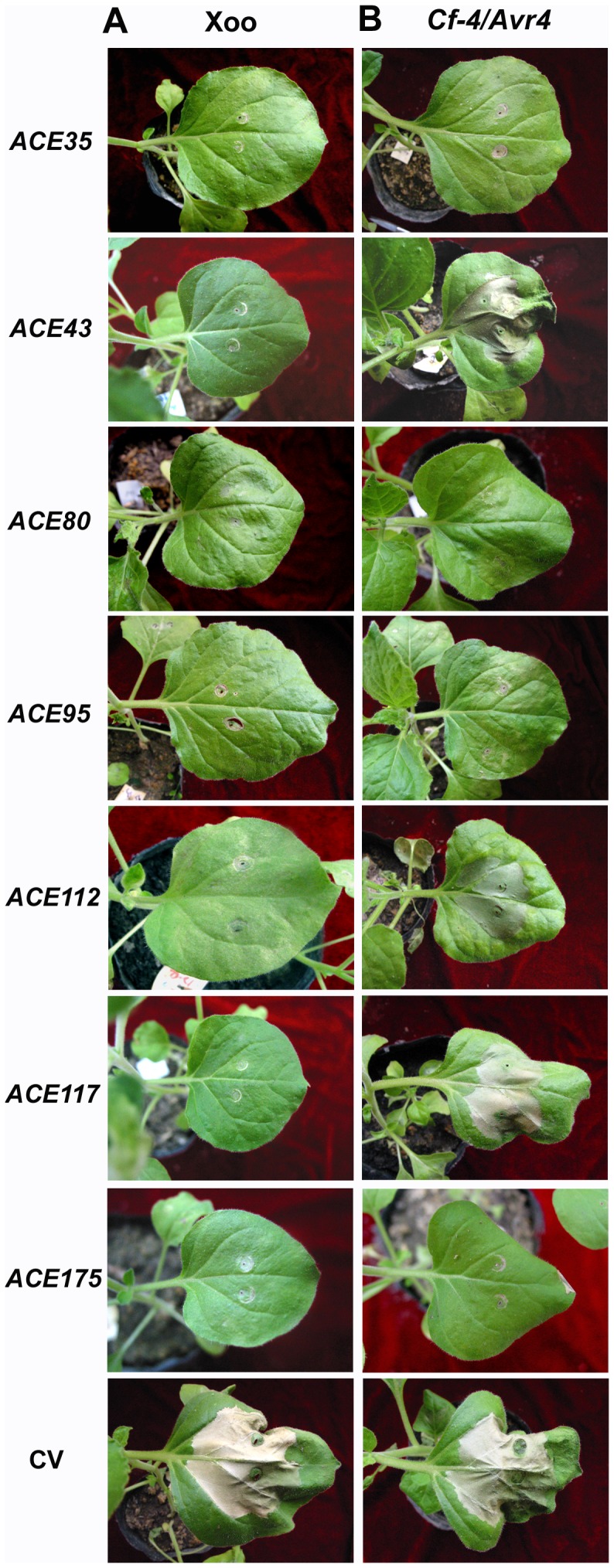
Effect of silencing of seven *ACE* genes on Xoo-induced HR (A) and Cf-4-dependent HR (B) in *N. benthamiana.* HR assays was performed three weeks after agroinfiltration. Control vector (CV, containing a fragment of eGFP gene in pTRV2)-agroinfiltrated plants were used as control plants. Photographs were taken at 3 dpi.

In VIGS-treated plants, the severity of the HR varied depending on the gene subjected to silencing. When silenced, most of the 45 *ACE* genes did not significantly alter the HR in response to Xoo (data not shown). However, silencing of seven *ACE* genes (*ACE35*, *ACE43*, *ACE80*, *ACE95*, *ACE112*, *ACE117* and *ACE175*) either abolished or compromised the HR. No or only partial HR phenotype was observed in 66%∼80% of the silenced plants depending on the gene for silencing (Table 1; Figure 5A). These results indicate that the seven *ACE* genes are required for the HR in the Xoo-*N. benthamiana* interaction.

**Table 1:** Effect of silencing of seven *ACE* genes on Xoo-induced HR in *N. benthamian.*

*ACE* genes for silencing	VIGS experiment	HR^+^ leaves/Totalinfiltrated leaves^a^	Percentage HR^+^leaves (%)	Average percentage HR^+^leaves ± SE
CK	1	46/48	95.8	93.3±3.0
	2	47/50	94.0	
	3	36/40	90.0	
*ACE 35*	1	15/44	34.1	29.2±4.3
	2	12/46	26.1	
	3	11/40	27.5	
*ACE 43*	1	14/44	31.8	28.6±3.1
	2	12/47	25.5	
	3	10/35	28.6	
*ACE 80*	1	18/48	37.5	33.7±3.9
	2	17/50	34.0	
	3	11/37	29.7	
*ACE 95*	1	9/48	18.8	17.9±2.6
	2	10/50	20.0	
	3	6/40	15.0	
*ACE 112*	1	13/50	26.0	31.0±4.6
	2	16/50	32.0	
	3	14/40	35.0	
*ACE 117*	1	15/48	31.3	27.9±3.2
	2	12/48	25.0	
	3	11/40	27.5	
*ACE 175*	1	9/50	18.0	19.8±3.6
	2	11/46	23.9	
	3	7/40	17.5	

a HR^+^ leaves here refer to Xoo-infiltrated leaves showing full and nearly full HR phenotype (Figure S2).

**Figure 6:**
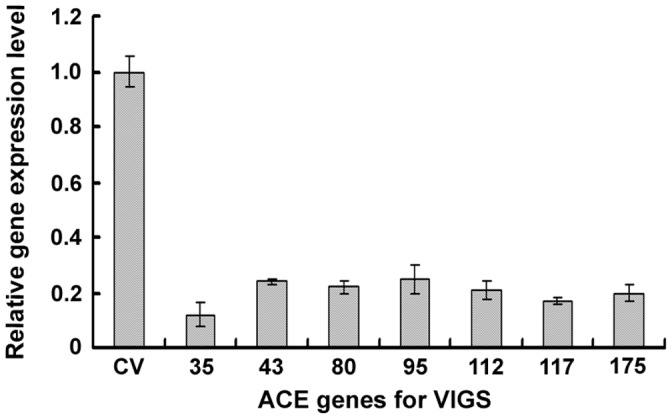
Efficiency of *ACE* gene silencing in *N. benthamiana* plants. Gene silencing efficiency was evaluated with the change of transcript abundance of genes for silence three weeks after agroinfiltration in silencing-treated plants in comparison with that in CV-treated control plants. Transcript abundance of target genes was analyzed by qRT-PCR. A 18s rDNA gene was also analyzed as inner loading control.

To verify the efficiency of *ACE* gene silencing, bioinformatic analysis was conducted to identify possible genetic targets of the seven *ACE* genes in *N. benthamiana*. BLASTn analysis of the recently released *N. benthamiana* draft genome and unigene databases (http://solgenomics.net/) revealed the existence of *N. benthamiana* homologs of the seven *ACE* genes (Table S2). Homologous sequences corresponding to *ACE35*, *ACE43*, *ACE117* and *ACE175* belonged to a small gene family (Table S2). To analyze silencing efficiency in *N. benthamiana*, transcript accumulation was monitored in the silenced plants by qRT-PCR using primers derived from *N. benthamiana* homologs of the tomato *ACE* genes. When a corresponding gene family was available, the conserved regions within related genes were used to design primers so that the entire family could be silenced (Table 2). As shown in Figure 6, transcripts of *N. benthamiana* homologs of the *ACE* genes in silenced tissues accumulated to much lower levels (<25%) as compared to CV controls, demonstrating that *ACE* gene silencing was efficient.

**Table 2:** PCR primers used in this study.

Gene	GenBank/SOL Accession no.	Sequence (Forward primer/Reverse primer, 5′→3′)	PCR product (bp)
*PR1*	X06930	GTGCCCAAAATTCTCAACA/AAATCGCCACTTCCCTCAG	196
*PR2*	M60460	TTTGATGCCCTTTTGGATTC/CTGCCCCGCTTTTCACAT	172
*PR4*	AF154635	TGGGTGGACATATTACAGAG/GGCACGCCGACACATTT	181
*PR5*	AF154636	GTGGGCGCCCTGGAAGAGT/CACGCGACAGTACATAAAAGTT	183
*NPR1*	DQ837218	CGCCGGCGGAGATTACTTCACT/GGACTCCTCGCCGACAAAATG	163
*HSR203J*	X77136	AGGCGGCGGCTTTTGTGTCA/GAGAGGTCCCGGAGCCAGAGG	180
*HIN1*	AB091429	TTCCGCCACCAGCAAAATC/TTAGGACGAAGAACGAGCCATA	166
*ACE35*	SGN-U506555/SGN-U506554/SGN-U514099/SGN-U518716	CTGAGCATAAACCAAGTAG/TCTCAGATCCTGAAGAAG	84
*ACE43*	Niben.v0.3.Scf25259845/Niben.v0.3.Scf25072334	AGAAGAGGGTGGATAATGGC/TCAAATTCAAGGCGGCGGCA	91
*ACE80*	Niben.v0.3.Scf25259834	AGAAATGGACCTCCTACCTATGA/GGATCTGCCTTATGGACTATTTC	143
*ACE95*	Niben.v0.3.Scf25288556	ACTTCACATCAAGATAG/TCTCAACACAAACAGGT	174
*ACE112*	Niben.v0.3.Scf25244033	CCTCCGGAAGCACCAAAATC/CAGAAATGAAGACGAATGTAAT	195
*ACE117*	Niben.v0.3.Scf25290305/Niben.v0.3.Scf24806883	GTTGATACATATTACAGAAAAG/TTGCTTCTCTTGTACAGACTGG	109
*ACE175*	SGN-U515867	AAGTTTTGACGGGGAATAC/CCGGTGAGGGAGGCAAGTT	123
*18s-rDNA*	AJ236016	AGGATTGACAGACTGAGAGC/CACAGACCTGTTATTGCCTC	210

### Genes Required for Xoo-mediated HR are also Essential for Nonhost Resistance

**Figure 7:**
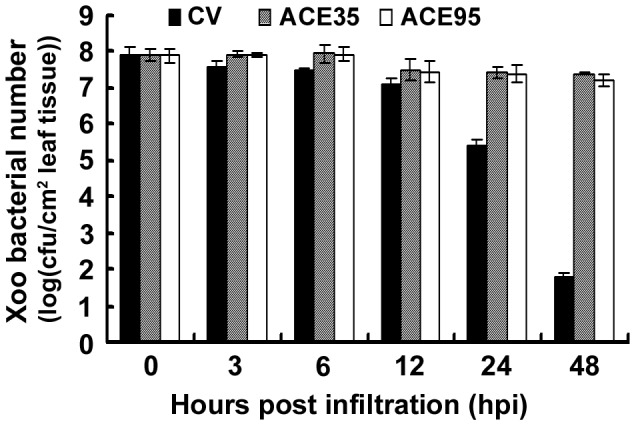
Effect of *ACE95* and *ACE35* gene silencing on nonhost resistance to Xoo in *N. benthamiana* plants. Bacterial cell numbers of Xoo in the infiltrated leaf tissues of *ACE95*- and *ACE35-*silenced plants and CV control plants was determined based on calculation of colony-forming units of the sampled tissues.

Xoo cell numbers in the infiltrated leaves of silenced and CV plants were compared. In CV plants, hypersensitive necrosis was apparent at 24 hpi (Figure 5A), which is typical for wild-type plants (Figure 1). Bacterial numbers in infiltrated leaves of CV plants decreased dramatically during the HR (e.g. 2.5- and 6.1-order of magnitude decrease at 24 and 48 hpi, respectively; Figure 7). However, the HR was either weak or nonexistent in *ACE35*- and *ACE95*-silenced plants (Figure 5A), and bacterial numbers continued to slowly increase (Figure 7). Bacterial numbers in the *ACE35*- and *ACE95*-silenced plants were significantly higher than CV plants beginning at 24 hpi (2- and 5-order of magnitude increase at 24 and 48 hpi, respectively), which negatively correlated with the profoundity of HR in the silenced plants (Table 1; Figure 7). Results obtained for the remaining five *ACE* genes were similar to *ACE35* and *ACE95* (data not shown). These data further proved the efficiency of silencing in this study. The results confirmed that the necrosis mediated by Xoo infiltration is a type of HR and demonstrated that *ACE* genes were essential for nonhost resistance to Xoo in *N. benthamiana*.

### Regulatory Pathways for the HR and Nonhost Resistance to Xoo Partially Overlap with those of Cf-dependent HR

We compared the HR produced in response to the bacterial pathogen Xoo with that elicited in response to a fungal pathogen (e.g. the *C. fulvum* effector Avr4 and the tomato R protein Cf-4). The seven *ACE* genes that were required for the HR to Xoo were silenced by VIGS to investigate their role in the Cf-4/Avr4-dependent HR. Results showed that silencing of *ACE35*, *ACE80*, *ACE95* and *ACE175*, but not *ACE43*, *ACE112* and *ACE117*, compromised Cf-4/Avr4-dependent HR, and led to either complete suppression of the HR or a partial HR in 70% of the silenced plants (Table 3; Figure 5B). These results demonstrated that *ACE35*, *ACE80*, *ACE95* and *ACE175* were required for the HR in both the Xoo/*N. benthaminana* and Cf-4/Avr4 interactions, thus indicating that the regulatory pathways for the HR in both interactions partially overlapped.

**Table 3:** Effect of silencing of seven *ACE* genes on Cf-4/Avr4-dependent HR in *N. benthamian.*

*ACE* genesfor silencing	VIGS experiment	HR^+^ leaves/Totalinfiltrated leaves^a^	Percentage HR^+^leaves (%)	Average percentage HR^+^leaves ± SE
CK	1	40/43	93.0	94.5±2.3
	2	42/45	93.3	
	3	35/36	97.2	
*ACE 35*	1	12/44	27.3	28.8±2.4
	2	12/38	31.6	
	3	11/40	27.5	
*ACE 43*	1	37/40	92.5	88.0±4.0
	2	39/46	84.8	
	3	33/38	86.8	
*ACE 80*	1	10/40	25.0	28.7±3.7
	2	12/37	32.4	
	3	10/35	28.6	
*ACE 95*	1	14/50	28.0	26.9±1.7
	2	12/48	25.0	
	3	10/36	27.8	
*ACE 112*	1	40/48	83.3	86.9±3.4
	2	45/50	90.0	
	3	35/40	87.5	
*ACE 117*	1	44/50	88.0	87.7±2.5
	2	40/47	85.1	
	3	36/40	90.0	
*ACE 175*	1	8/48	16.7	20.4±4.2
	2	11/44	25.0	
	3	7/36	19.4	

a HR^+^ leaves here refer to agroinfiltrated leaves showing full and nearly full Cf-4/Avr4-dependent HR phenotype (Figure S2).

### Annotation of *ACE* Genes Required for the HR in Response to Xoo and Cf-4/Avr4 Interaction

Thanks to the public availability of the tomato genome, annotation of the *ACE* genes could be updated. Based on our cloned *ACE* EST sequences, bioinformatic analyses were conducted using the tomato genome (http://solgenomics.net/), the Uniprot protein database (http://www.uniprot.org/) and the Pfam domain tool (http://pfam.sanger.ac.uk/, release 25.0). This resulted in the identification of full-length *ACE* genes, functional domains, and functional classification (Table 4). Data mining revealed that *ACE35*, a previously unknown gene, encoded a calreticulin protein. Calreticulin is a conserved calcium-binding protein that is involved in more than 40 cellular processes such as calcium storage and signaling, chaperone activity, cell adhesion, and regulation of gene expression [32]. It functions in many biological processes, including plant-pathogen interactions [33]. *ACE43* encoded an ethylene-responsive transcription factor (ERF), which regulates the expression of GCC-box-containing genes, including a set of defense-related genes [34]–[35]. *ACE80* encoded a protein of unknown function; however, it contained PB001238, a Pfam-B domain that is conserved in 91 sequences in diverse prokaryotic and eukaryotic organisms. Although this group of sequences shows similarity to genes encoding PR proteins with unknown biological property, ACE80 was not related to known Solanaceous PR proteins (Uniprot database; data not shown). Thus ACE80 may be a novel Solanaceous PR protein. Bioinformatic analysis revealed that ACE117 was a hydrolase with an alpha/beta fold, ACE175 was a peroxidase, and ACE95 and ACE112 had unknown functions (Table 4).

**Table 4:** Annotation of the seven *ACE* genes required for HR and nonhost resistance to Xoo and/or Cf-4/Avr4-dependent HR.

ACE gene	Corresponding SGNgeneor locus name	Product	Pfam/InterPro domain	Functional class
175	Solyc02g079500.2	Peroxidase	Peroxidase (PF00141/IPR002016)	Defense-related
80	Solyc04g064880.2	Novel Solanaceous protein,probably pathogenesis-relatedfamily protein	PB001238	Probably defense-related
43	Solyc12g056590.1	Ethylene responsivetranscription factor (ERF)	AP2 (PF00847/IPR001471)	Transcriptional regulator
35	Solyc04g048900.2	Calreticulin calcium-bindingprotein	Calreticulin (PF00262/IPR001580), PB003966	Miscellaneous
117	Solyc01g103650.2	Hydrolase alpha/beta foldfamily	Abhydrolase_6 (PF12697/IPR012020)	Uncertain
95	Unigene SGN-U600587	Unknown protein	−	Unknown
112	Solyc01g096620.2	Unknown protein	−	Unknown

Collectively, our data reveal that the proteins ACE35 (calreticulin), ACE80 (novel Solanaceous protein), ACE175 (peroxidase) and ACE95 (unknown function) are required for nonhost resistance to Xoo and the Cf-4/Avr4-dependent HR. ACE43 (ERF), ACE117 (hydrolase), and ACE112 (unknown function) are required for nonhost resistance to Xoo but are dispensable for the Cf-4/Avr4-dependent HR.

## Discussion

### A Convenient, Efficient System for Screening Genes Required for Nonhost Resistance

Although the potential of nonhost resistance in protecting crops from plant diseases is well-documented, the underlying mechanism of nonhost resistance to Xoo, which causes a serious disease of rice, remains unclear. One aim of this study was to establish an efficient system to screen for genes required for nonhost resistance to Xoo. Thus it was essential to identify a suitable plant that was a nonhost of Xoo, and *N. benthamiana* was selected based on several considerations. *N. benthamiana* is easily grown, produces seed quickly, is amenable to manipulation, and a draft of the genome has been recently released (http://solgenomics.net/). The pathogen Xoo has a narrow host range that consists of rice, several species of wild rice, and a number of graminaceous weeds [11]. Thus, the plants infected by Xoo are not related to *N. benthamiana* and the latter can be regarded as a nonhost of Xoo. Inoculation of a given pathogenic bacterium into nonhost plants is generally accompanied by nonhost resistance and a macroscopically visible HR [36]–[37].

Tobacco (*N. tabacum*) is a popular plant for HR assays, since it is easy to infiltrate with bacteria and a readily visible hypersensitive necrosis occurs when resistance is manifested [38]–[39]. *N. benthamiana* is phylogenetically related to *N. tabacum* and shares many of the same favorable characteristics for nonhost resistance studies. Indeed, we found that infiltration of Xoo into *N. benthamiana* plants was a simple process, and hypersensitive necrosis was macroscopically visible within 24 hpi (Figure 1). More importantly, this visible HR is a hallmark of nonhost resistance to Xoo (Figs. 2, 3, 4), and thus is a nice nonlaborious report trait for genetic screening for genes required for this nonhost resistance. Furthermore, *N. benthamiana* is the plant of choice for VIGS, which is a powerful, reverse genetic tool to analyze gene function. VIGS analysis is rapid, does not require plant transformation, and can be used for genes where only partial sequences are available [40]–[41]. Thus, VIGS can be used to functionally screen genes with potential roles in nonhost resistance. In the present study, we used VIGS and an HR detection assay to screen for genes involved in nonhost resistance in the Xoo/*N. benthamiana* interaction. Although our assay was developed for Xoo, it should be suitable for detecting nonhost resistance to other microbes that elicit an HR in *N. benthamiana*.

### Nonhost Resistance to Xoo: Involvement of ROS and Calcium-dependent Pathways

We used our screening system and previously identified *ACE* (Avr/Cf- elicited) genes [30]–[31] to identify seven genes that are required for the HR and nonhost resistance to Xoo. Silencing of these genes abolished or markedly compromised the HR and nonhost resistance (Table 1, Figs. 5A, 7). Notably, four of the seven genes were required for Cf-4/Avr4-dependent HR [30]–[31]. Thus, regulatory pathways of nonhost resistance to the bacterial pathogen Xoo and those of ETI (Cf-4/Avr4) partially overlap, which supports the hypothesis that nonhost and host resistance are mechanistically similar [8].

Among the genes identified in this study, *ACE43* and *ACE175* are known to be involved in defense. *ACE43* encodes an ethylene-responsive transcription factor, which regulates gene expression by binding to ethylene-responsive GCC box elements in the promoters of defense-related genes, including *PR* genes and ethylene-, and jasmonic acid-induced genes [34]–[35]. *ACE175* encodes a peroxidase (POD). A family of POD isoenzymes participate in a variety of physiological processes, including lignification, suberization and other defense mechanisms [42]. More importantly, PODs in the apoplastic space catalyze the generation of ROS, which are coupled to the oxidation of defense signaling compounds such as salicylic acid (SA), indole-3-acetic acid (IAA), aromatic monoamines (AMAs) and chitooligosaccharides (COSs) [43]. We discovered that H_2_O_2_, an important ROS, accumulated to high levels prior to hypersensitive necrosis in Xoo-infiltrated leaves (Figure 2). To probe the contribution of the putative peroxidase encoded by *ACE175*, we evaluated the effect of silencing of this gene on ROS accumulation. There was no significant difference in ROS accumulation as detected by DAB staining after Xoo infiltration in silenced or nonsilenced plants (data not shown). Thus *ACE175* does not play a pivotal role in ROS accumulation. Alternatively, other pathways may exist to compensate for the ACE175 POD-dependent pathway, such as the recently-identified peroxisomal glycolate oxidase (GOX)-dependent pathway [44].

VIGS analysis revealed that *ACE35* is required for both the HR and nonhost resistance to Xoo and for Cf-4-dependent HR. *ACE35* encodes a calreticulin, which is a conserved, calcium-binding protein that stores calcium in plants [45]. Calreticulins are involved in a variety of cellular processes, including calcium signaling, chaperone activity, cell adhesion, and regulation of gene expression [32]. Calreticulin is believed to play a role in plant-pathogen interaction. Calreticulin gene expression is induced after exposure to elicitors [46] and during Cf-4-dependent HR [30]; furthermore, the protein is phosphorylated when tobacco cells were treated with elicitor-active oligogalacturonides [47]. Calreticulin was shown to co-localize with tobacco mosaic virus movement protein, and both proteins co-localized in the plasmodesmata of tobacco cells, implicating a role for calreticulin in plant-viral interactions [33].

To our knowledge, our results provide the first evidence that calreticulin plays an important role in the HR and nonhost resistance. Since calcium signaling components function in plant defense [48], these results raise interesting questions regarding the identification of calreticulin- and calcium-modulated pathways and effectors that regulate nonhost resistance to Xoo and Cf-4-dependent HR. Mitogen-activated protein kinase (MAPK) cascades and calcium-dependent protein kinases (CDPKs) are potential candidates [49]. It has been reported that MAPKs and a tobacco CDPK gene, *NtCDPK2*, are required for Cf-9/Avr9-dependent HR [50]–[51]. Additionally, two CDPKs (StCDPK4 and StCDPK5) phosphorylate NADPH oxidases and thereby positively regulate the production of ROS [52]. However, superoxide anion (O_2_^⋅−^), which generates mainly in a NADPH oxidase-dependent way, seemed not to be important in Xoo-triggered ROS accumulation process, although it actually is for Cf-dependent ROS generation. It will be interesting to investigate whether CDPKs affect regulatory proteins that control H_2_O_2_ generation and accumulation, such as the newly-identified GOX [44]. It has been reported that SA-, AMA- and COS- and POD-mediated generation of ROS increases the level of cytosolic calcium [43]. Although it is unclear whether the *ACE35*-encoded calreticulin is involved in calcium accumulation, our results indicate the potential existence of an amplification mechanism for ROS by a calcium-dependent signaling pathway in nonhost resistance.

ACE80 was required for the HR and nonhost resistance to Xoo and for Cf-4-dependent HR. According to Pfam analysis, ACE80 contains the Pfam-B domain PB001238, which is conserved in 91 sequences from 51 prokaryotic and eukaryotic organisms. Among these are 45 plant sequences, including some that are annotated as pathogen- or pathogenesis-related proteins. However, ACE80 has no significant sequence similarity to 17 families of well-known PR proteins [53]. Although the 45 conserved sequences were derived from 14 plant species, Solanaceous species were not represented. Thus ACE80 is a newly-identified Solanaceous protein that is required for both HR and nonhost resistance.

The seven genes identified in this study provide a preliminary working model for the HR and nonhost resistance to Xoo and Cf-4-dependent HR. An oxidative burst and calcium surge are critical components of the signaling pathways leading to HR and nonhost resistance in *N. benthamiana*. The apoplastic ACE175 POD may catalyze the generation of ROS that is coupled to SA oxidation; this may result in elevated calcium levels via the ACE35 calreticulin. The calcium signal is potentially transduced into a phosphorylation signal by activating the MAPK cascade and CDPKs. CDPKs then phosphorylate plasma membrane NADPH oxidase and other components that function in the oxidative burst, resulting in release of more ROS and forming an amplification loop for ROS generation. ROS and calcium signals further activate downstream components, which might contain the ACE43 ERF, ACE117 hydrolase, ACE80, ACE95, and ACE112, thereby resulting in HR and nonhost resistance to Xoo. Elements of the Cf-4-dependent HR partially overlap with the above pathways and share the ROS oxidative burst, calcium signals, and several downstream components (e.g. ACE80 and ACE95). This working model is derived from the genes identified in this study and from results obtained for other plant pathosystems. Although the 45 *ACE* genes were the basis for our screen, we acknowledge the existence of additional factors that were not identified in the present study. A broader screen will likely result in the discovery of other key regulatory proteins that function in nonhost resistance. Furthermore, functional dissection of the seven genes identified in this study will provide new insights into underlying molecular basis of nonhost resistance.

## Materials and Methods

### Infiltration of *N. benthamiana* with Xoo and the *ΔhrcU* Mutant

Xoo strain YN-1 and the *ΔhrcU* mutant were grown at 28°C in nutrient agar (NA) containing the following reagents in g/L: sucrose, 10; polypeptone, 5; yeast extract, 1; beef extract, 3; and Bacto agar, 15; pH 7.0–7.2. Single colonies were transferred to nutrient broth (NB) with agitation until the OD_600_ was 0.5. Bacterial cells were collected by centrifugation at 8000× g for 5 min, washed three times with sterile dH_2_O, and suspended at 8×10^7^ cfu/ml. Bacterial cells were then infiltrated into leaves of *N. benthamiana* using a needleless syringe. Inoculated plants were maintained in plant growth chambers at 26–28°C with a 16/8 h light/dark photoperiod. Inoculated plants were examined at various times after inoculation (0, 12, 24, 48 hpi) and photographed.

### Determination of Bacterial Numbers Inside Plant Leaves

Bacterial numbers in Xoo-infiltrated leaves of wild-type, control, and silenced plants were determined as reported previously [54].

### Histochemical Detection of H_2_O_2_ and O_2_^⋅−^

Leaves that were infiltrated with bacterial cells or sterile H_2_O were sampled at 0, 3, 6, 12, 24 and 48 hpi. H_2_O_2_ was detected *in situ* using DAB staining as described previously [2], inspected using light microscopy, and photographed. O_2_^⋅−^ was detected by NBT staining [55].

### Expression Analysis of HR- and Defense-related Genes

Transcription of *HIN1* and *HSR203J* (HR marker genes), *NPR1*, and *PR* genes was analyzed using qRT-PCR and RT-PCR. The Xoo-infiltrated area was sampled at 0, 3, 6, 12, 24 and 48 hpi. Total RNA was extracted with TRIzol (Invitrogen, USA), and reverse transcription from total RNA was conducted using the PrimeScript RT-PCR kit (TaKaRa Biotechnology, China). PCR was then performed from cDNAs using gene-specific primers (Table 2). The StepOne Real-Time PCR system (Applied Biosystems, USA) and SYBR Green PCR Master Mix (TaKaRa) were used for qRT-PCR analysis. 18s rDNA was used as internal control. The primers used for PCR are listed in Table 2. The relative expression of target genes was calculated based on a value of 2^−ΔΔCt^ as recommended by the manufacturer. PCR products obtained from RT-PCR were analyzed by agarose gel electrophoresis.

### VIGS Screening Analysis

*ACE* (Avr/Cf elicited) cDNA fragments were cloned previously in pUCm-T [30]–[31]. Forty five were used in the VIGS screen for potential regulators of the HR and nonhost resistance in the Xoo/*N. benthamiana* interaction. These fragments were subcloned into the TRV VIGS vector pYL156 as *Eco*RI/*Bam*HI or *Eco*RI/*Xho*I fragments. TRV-induced gene silencing for these genes in *N. benthamiana* plants was conducted as described previously [56]–[57]. Silencing of the *N. benthamiana* phytoene desaturase gene (*NbPDS*) was conducted to check the efficiency of the VIGS procedure. Three weeks after agroinfiltration (when the genes were silenced completely), the plants were subjected to HR assays. For each gene, at least 10 plants were used for each VIGS experiment; and studies were conducted in triplicate.

VIGS efficiency was evaluated by comparing transcript abundance in silenced plants with those of the control plants (CV) three weeks after agroinfiltration. Transcript abundance of target genes was analyzed by qRT-PCR as described above using the gene-specific primers listed in Table 2.

### HR Analysis

For *Cf-4/Avr4-*dependent HR, *Agrobacterium tumefaciens* expressing interacting *Avr* and *Cf* genes were cultured as described [56]. Bacterial cells were collected by centrifugation and suspended at an OD_600_ of 4.0. Suspensions of *A. tumefaciens* expressing an *Avr* gene and its complementary *Cf* gene were mixed in a 1∶1 ratio to obtain the final ‘agro-inoculum’ for infiltration. Agro-inocula were infiltrated into three apical expanded leaves of *N. benthamiana* plants with a sterile, needleless syringe. The agro-inoculated plants were maintained in plant growth chambers at 25°C with a 16/8 h light/dark photoperiod. Three days later, the HR in the inoculated area was investigated and photographed.

For HR assays in response to Xoo, bacterial cells were adjusted to a concentration of 8×10^7^ cfu/ml and infiltrated into plant leaves as described above for *Cf-4/Avr4.*

### Updated Annotation of *ACE* Genes

Our previously cloned *ACE* fragments were used to search the tomato genome (http://solgenomics.net/) using BLASTn. Full-length genes were then analyzed using the Uniprot database (http://www.uniprot.org/) and the Pfam domain tool (http://pfam.sanger.ac.uk/, release 25.0) to obtain information about protein function and conserved domains.

## Supporting Information

Figure S1 **Upregulation of HR- and defense-related genes in Xoo-infiltrated leaves.** Gene expression was analyzed by RT-PCR with gene specific primers. HR marker genes *HIN1* and *HSR203J*, a pivatol defense regulator *NPR1* and a set of *PR* genes were analyzed for their expression in Xoo-infiltrated plants at 12 hpi and 24 hpi.
(TIF)

Figure S2 **Phenotypes of Xoo-infiltrated leaves of the silencing-treated** ***N. benthamiana*** **plants.** Xoo suspension at 8×10^7^ cfu/ml was infiltrated into leaves of silencing-treated plants. Photographs were taken at 3 dpi.
(TIF)

Table S1 **List of the 45** ***ACE*** **genes selected for VIGS functional analysis.**
(DOC)

Table S2 **Bioinformatics analysis of** ***N. benthamiana*** **homologs of tomato** ***ACE*** **genes.** *N. benthamiana* orthologs of the tomato *ACE* genes were searched on *N. benthamiana* draft genome database and unigene database (http://solgenomics.net/) using BLASTn program.
(DOC)
