# Supplementary figures and images for: Identification of Genes Required for Nonhost Resistance to Xanthomonas oryzae pv. oryzae Reveals Novel Signaling Components
Source: PLoS One. 2012 Aug 13;7(8):e42796. doi: 10.1371/journal.pone.0042796 (PMC3418293; doi:10.1371/journal.pone.0042796)

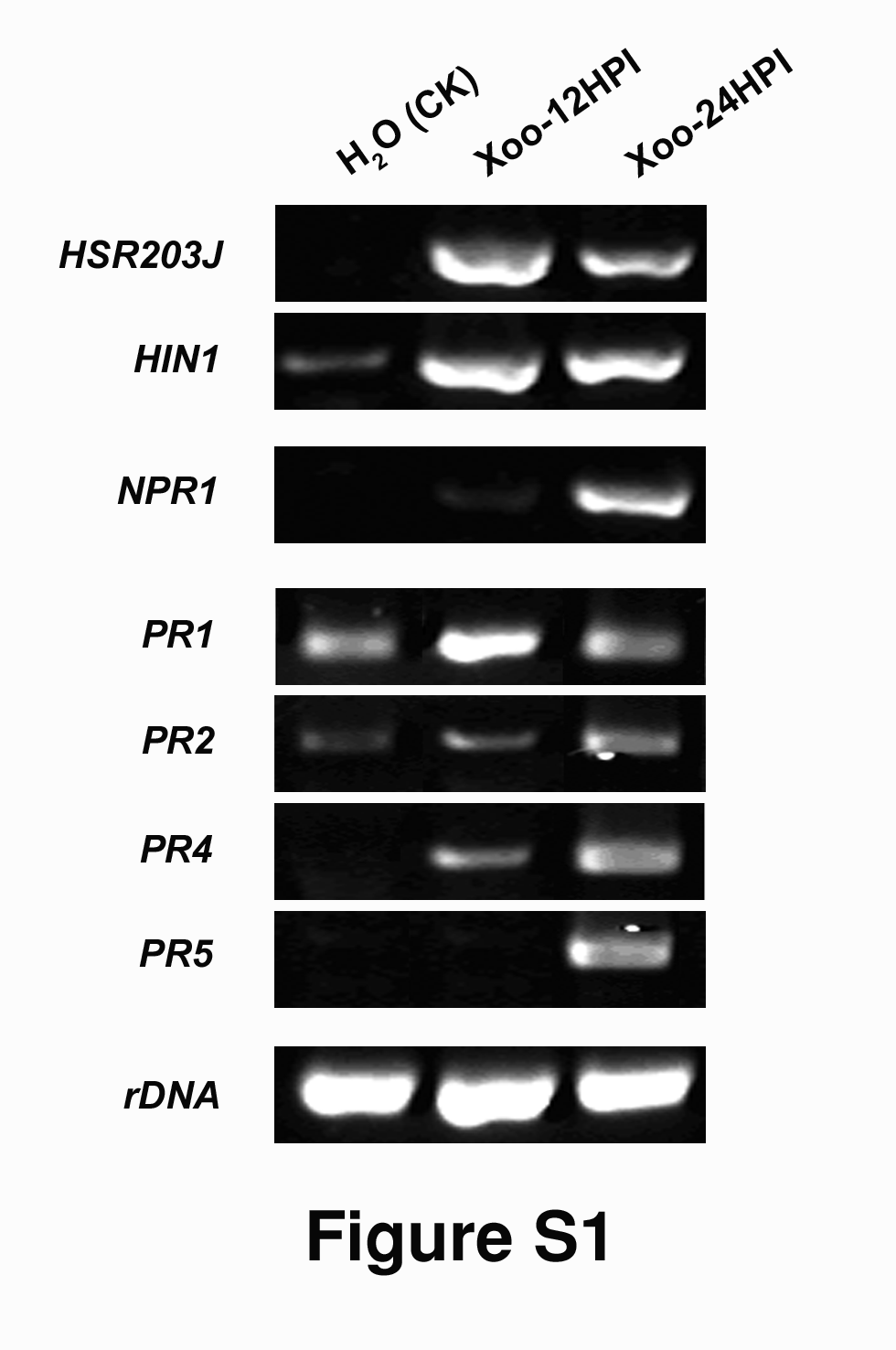

Supplement: Figure S1 — Upregulation of HR- and defense-related genes in Xoo-infiltrated leaves. Gene expression was analyzed by RT-PCR with gene specific primers. HR marker genes HIN1 and HSR203J, a pivatol defense regulator NPR1 and a set of PR genes were analyzed for their expression in Xoo-infiltrated plants at 12 hpi and 24 hpi. (TIF) [file pone.0042796.s001.tif]

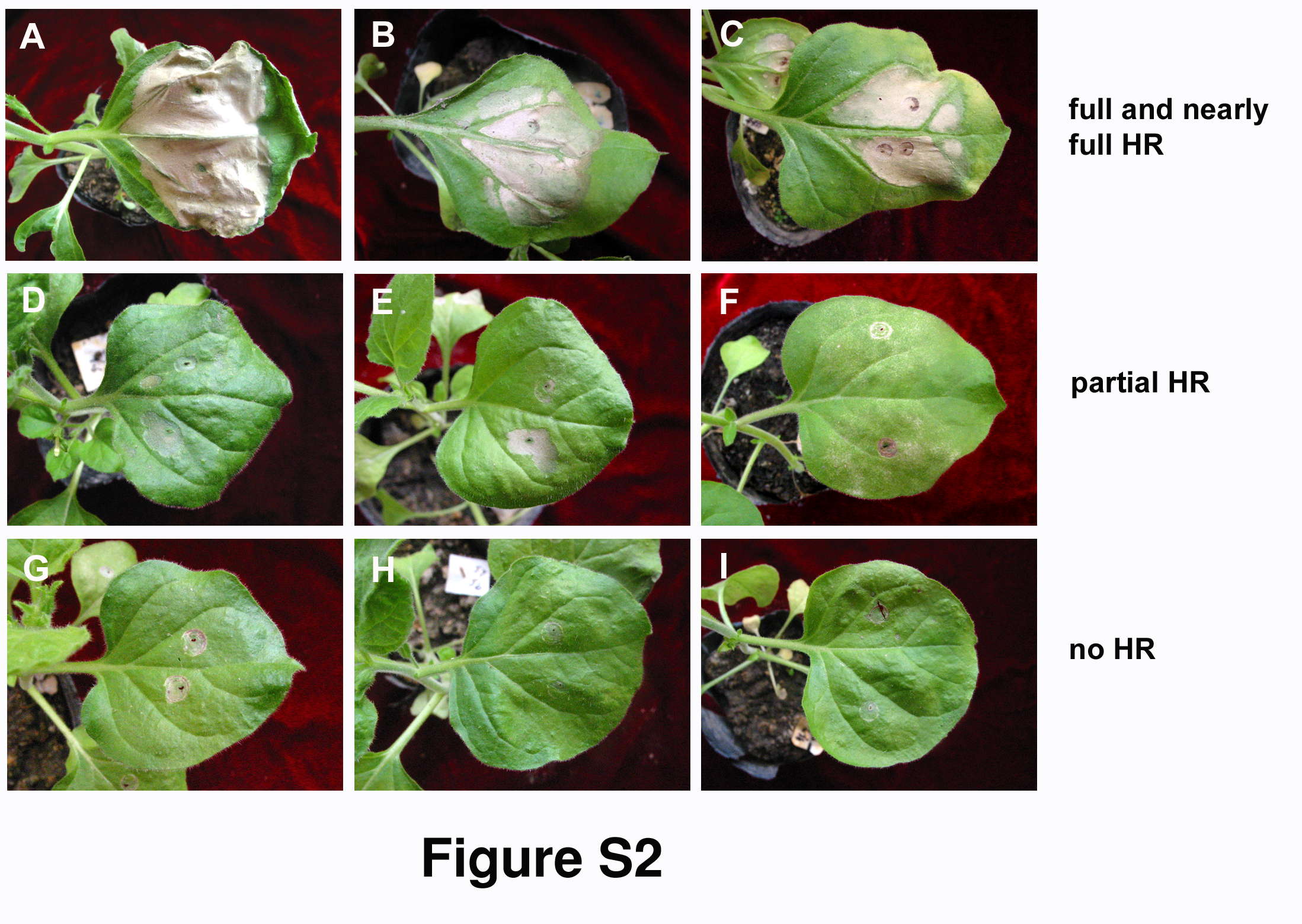

Supplement: Figure S2 — Phenotypes of Xoo-infiltrated leaves of the silencing-treated N. benthamiana plants. Xoo suspension at 8×10^7^ cfu/ml was infiltrated into leaves of silencing-treated plants. Photographs were taken at 3 dpi. (TIF) [file pone.0042796.s002.tif]
