# Supplementary material for: Identification of Genes Required for Nonhost Resistance to Xanthomonas oryzae pv. oryzae Reveals Novel Signaling Components
Source: PLoS One. 2012 Aug 13;7(8):e42796. doi: 10.1371/journal.pone.0042796 (PMC3418293; doi:10.1371/journal.pone.0042796)
Supplement: Table S1 — List of the 45 ACE genes selected for VIGS functional analysis. (DOC) [file pone.0042796.s003.doc]

**Table S1 List of the 45 *ACE* genes selected for VIGS functional analysis**

| **Functional class** | **Product** | ***ACE* fragment** | **GenBank accession number** |
| --- | --- | --- | --- |
| HR / cell death  associated | Kunitz-like endopeptidase inhibitor | 33 | CD579117 |
|  |  | 168 | CK348331 |
|  | Hsr203J-like protein | 111 | CK348296 |
| Defense-related | Subtilisin-like proteinase | 150 | CK348322 |
|  | Subtilisin-like proteinase | 189 | CK348341 |
|  | Protease inhibitor /lipid transfer protein (LTP) family | 225 | CK348363 |
|  | Suberization-associated anionic peroxidase 1 precursor | 175 | CK348380 |
|  | Lipid transfer protein | 173 | CK348379 |
|  | NtEIG-E80 protein | 154 | CK348325 |
|  | Proteinase inhibitor II | 108 | CD579161 |
|  | ABC transporter | 38 | CD579122 |
|  | Lipid transfer protein | 68 | CD579139 |
|  | Pathogenesis-related family protein | 80 | CD579145 |
| Signaling-related | Calmodulin-like protein | 146 | DN956215 |
|  | Calcium binding protein /calmodulin-related protein 2, touch-induced (TCH2) | 170 | CK348332 |
|  | Lipoxygenase | 202 | CK348347 |
|  | Leucine-rich repeat transmembrane protein kinase | 41 | CD579124 |
|  | Shaggy-related protein kinase eta | 71 | CD579142 |
|  | Protein kinase family protein AtSIK | 169 | DN956218 |
|  | Yeast pheromone receptor protein AR781 | 119 | CK348303 |
|  | 1-aminocyclopropane-1-carboxylate oxidase | 164 | DN956216 |
|  | Pyrophosphate phospho-hydrolase | 4 | CK348287 |
| Transcriptional  regulator | ERF (ethylene response factor) subfamily B-4 of ERF/AP2 transcription factor family | 43 | CD579126 |
|  | WRKY transcription factor | 145 | CK348319 |
|  | Zinc finger (C2H2 type)-containing DNA/RNA binding protein | 231 | CK348369 |
|  | Yippee zinc-binding family protein | 32 | CD579116 |
| Protein transport | Tic62 protein precursor | 232 | CK348370 |
| Metabolism | Oxidoreductase, zinc-binding dehydrogenase family | 1 | CK348286 |
|  | Inosine-uridine nucleoside N-ribohydrolase | 138 | CK348313 |
|  | Glycosyltransferase | 149 | CK348321 |
|  | AMP-binding enzyme / probable acyl-CoA ligase medium chain | 191 | CK348342 |
|  | Cysteine synthase, chloroplast/O-acetylserine (thiol)-lyas /O-acetylserine sulfhydrylase | 234 | CK348371 |
| Miscellaneous | DnaJ-like protein / heat shock protein binding/ tuber-induction protein | 94 | CD579153 |
|  | ATP-dependent Clp protease ATP-binding subunit clpA homolog CD4A | 155 | CK348326 |
|  | Cytochrome P450 76A2 | 134 | CK348311 |
|  | cytochrome P450 | 229 | CK348367 |
|  | Glycine rich protein precursor | 45 | CD579127 |
|  | Hydrolase alpha/beta fold family / Embryogenesis-associated protein-related | 117 | CK348301 |
| Water transport | Major intrinsic family protein/ PM28B protein/ water channel protein | 240 | DN956225 |
| Unknown | Unknown protein | 35 | CD579119 |
|  | Unknown protein | 66 | CD579137 |
|  | Unknown protein | 95 | CD579154 |
|  | Unknown function | 112 | CK348297 |
|  | Unknown function | 227 | CK348365 |
|  | Expressed protein | 245 | CK348375 |
