# Supplementary material for: Identification of Genes Required for Nonhost Resistance to Xanthomonas oryzae pv. oryzae Reveals Novel Signaling Components
Source: PLoS One. 2012 Aug 13;7(8):e42796. doi: 10.1371/journal.pone.0042796 (PMC3418293; doi:10.1371/journal.pone.0042796)
Supplement: Table S2 — Bioinformatics analysis of N. benthamiana homologs of tomato ACE genes. N. benthamiana orthologs of the tomato ACE genes were searched on N. benthamiana draft genome database and unigene database (http://solgenomics.net/) using BLASTn program. (DOC) [file pone.0042796.s004.doc]

**Table S2 *Nicotiana benthamiana* homologs of the *ACE* genes**

| **ACE gene** | **Length (bp)** | **Nb genome/unigene homolog (BLASTn)** | **Identities** |
| --- | --- | --- | --- |
| 35 | 94 | SGN-U514099 | 49/94 |
|  |  | SGN-U518716 | 41/94 |
|  |  | SGN-U506555 | 49/94 |
|  |  | SGN-U506554 | 48/94 |
| 43 | 95 | Niben.v0.3.Scf25259845 | 66/95 |
|  |  | Niben.v0.3.Scf25072334 | 66/95 |
| 80 | 229 | Niben.v0.3.Scf25259834 | 201/229 |
|  |  | Niben.v0.3.Scf25223559 | 99/110 |
| 95 | 226 | Niben.v0.3.Scf25288556 | 140/302 |
|  |  | Niben.v0.3.Scf25281492 | 136/302 |
| 112 | 291 | Niben.v0.3.Scf25244033 | 187/228 |
|  |  | Niben.v0.3.Scf24928671 | 119/139 |
| 117 | 357 | Niben.v0.3.Scf25290305 | 164/357 |
|  |  | Niben.v0.3.Scf24806883 | 188/357 |
| 175 | 167 | Niben.v0.3.Scf25261138 | 77/167 |
|  |  | Niben.v0.3.Scf25214896 | 133/167 |
|  |  | Niben.v0.3.Scf25150635 | 133/167 |
|  |  | Niben.v0.3.Scf25209888 | 75/167 |
|  |  | Niben.v0.3.Scf25130179 | 81/167 |
